# Psychiatric care use among migrants to Sweden compared with Swedish-born residents: a longitudinal cohort study of 5 150 753 people

**DOI:** 10.1136/bmjgh-2020-002471

**Published:** 2020-09-24

**Authors:** Anna-Clara Hollander, Euan Mackay, Hugo Sjöqvist, James B Kirkbride, Sofie Bäärnhielm, Christina Dalman

**Affiliations:** 1 Global Public Health Sciences, Karolinska Institutet, Stockholm, Sweden; 2 Division of Psychiatry, Faculty of Brain Sciences, UCL, London, UK; 3 Clinical Neuroscience, Karolinska Institute, Stockholm, Sweden; 4 Transkulturellt Centrum, Stockholm Region, Stockholm, Sweden; 5 Centrum för epidemiologi och samhällsmedicin, Stockholm Region, Stockholm, Sweden

**Keywords:** epidemiology, mental health & psychiatry

## Abstract

**Background:**

To investigate differences in psychiatric care use over time between Swedish born and those born abroad who migrate to Sweden.

**Methods:**

Population-based cohort study analysing linked population and health registers, following individuals born 1944–1990 from 1 January 2005 to 31 December 2016. Time-stratified survival analysis using Cox regression estimated time to psychiatric care use. Population included 5 150 753 individuals with 78.1% Swedish born. Migrant status was coded as Swedish born or migrant. Migrants were grouped by year of immigration and region of origin. The main outcome: psychiatric care use, defined as any psychiatric care; psychiatric inpatient or outpatient care; or use of psychotropics.

**Results:**

Migrants arriving before 2005 had a higher use of any psychiatric care relative to Swedish born but migrants arriving 2005 onwards had lower use. Migrants from sub-Saharan Africa and Asia had a lower use of any psychiatric care during the first decade in Sweden whereas migrants from Middle East and North Africa had a higher use, driven by use of psychotropics.

**Conclusions:**

The lower use of psychiatric care during the first decade contrasts with higher use among migrants with a longer duration of stay. Psychiatric care use among migrants should be analysed multi-dimensionally, taking duration of stay, region of origin and type of care into account.

**Key questions**

**What is already known?**

- Migrants are subject to excess risks for developing mental illness compared with host populations.
- Migrants display lower psychiatric care utilisation than the general population.
- It remains unclear how these patterns are influenced by time in the new country, region of origin and type of psychiatric care, such as inpatient and outpatient care and psychotropic medication.

**What are the new findings?**

- The study, using high-quality register data, found that most migrants to Sweden typically underuse psychiatric services during the first decade in Sweden; however, use increases with time, and those who have been in Sweden more than a decade typically have higher use of psychiatric care than Swedish born.
- Migrant groups with a particularly low use during the first years were from sub-Saharan Africa, Asia, and Western and Southern Europe but migrants from the Middle East and North Africa had a higher psychiatric care utilisation since start of follow-up, driven by a higher use of psychotropic medication.

**What do the new findings imply?**

- Utilisation of psychiatric care among migrants is highly dependent on time in the country, with utilisation increasing with time.
- When studying psychiatric care utilisation in migrants, it is essential to include psychotropic medication as some groups of migrants are reliant on this type of psychiatric treatment during early immigration.
- Greater understanding of psychiatric care utilisation and access among migrants requires multidimensional analysis, taking duration of stay, region of origin and type of care into account.

## Introduction

Migrants are subject to excess risk of many mental disorders but not all; for instance, exhibiting higher rates of post-traumatic stress disorder (PTSD) and psychotic disorders than host populations.^1–3^ Despite this, migrant and ethnic minority populations in many high-income countries display divergent patterns of psychiatric care use from majority populations,^2^ subjected to greater compulsory detention,^4^ diminished use of outpatient services,^5^ less primary care involvement^6^ and a distinct profile of psychotropic medication use.^7 8^ Gender,^9^ ethnicity/region of origin^4 10 11^ and time in the new country^12^ are known to influence care use among migrants. Studies show that these skewed interactions with psychiatric care services are linked to barriers for migrants and ethnic minority populations in accessing mental healthcare, such as language issues,^13^ poor health literacy,^14^ culturally bound stigma of mental illness and discordant construction of mental health to host psychiatric system.^15^ However, understanding structural barriers such as institutional racism,^16^ geographic access to services^17^ and differential treatment by service providers is also essential.^18^

Using linked national registers of health and population data, psychiatric service use can be examined with high external validity, with findings generalisable for entire populations. A scoping review^2^ published in 2017 including studies from Sweden, Australia, Hong Kong, Croatia, Germany, Netherlands, Denmark, Brazil, Canada, Israel, Norway, Finland, Switzerland and USA concluded that migrants in high-income countries ‘utilise mental health services and psychotropic medication less than the settled majority population’, however noting that the studies typically lack length of stay in host country, and that only 4 of the 51 studies included had information on psychotropic medication. Psychiatric care use by migrants over time is poorly understood, as time in the host country is rarely taken into account in studies,^2^ and studies largely focus on either one type of care (eg, outpatient care or psychotropic medication), one diagnosis or one migrant group (eg, refugees).^2^ As different forms of psychiatric care have different applications and cannot be used interchangeably, understanding general psychiatric care use among migrants, as well as their use of inpatient and outpatient care and psychotropic medication, is essential to inform policy and ensure mental health needs in this complex vulnerable population are met.

Sweden has a total population of 10 million, with migrants representing almost one-fifth of this population as of 2018. Between 1940 and 1970 Sweden experienced high levels of labour immigration, followed by substantial refugee immigration peaking in 2015. As the Swedish healthcare system offers universal healthcare with small out-of-pocket costs, it offers great opportunities for register-based research. Migrants with a permanent residence permit are included in this system with no restrictions, allowing inquiry into their patterns of care use. The aim of this study is to investigate differences in patterns of psychiatric care use between people born in Sweden to two Swedish-born parents (henceforth ‘Swedish born’) and all migrants; and to determine how these patterns are influenced by time in Sweden, and region of origin. We hypothesise that migrants have a lower psychiatric care use than Swedish born during their first decade in Sweden, and that psychiatric care during the first decade is dominated by prescribed psychotropic medication, but that psychiatric care use increase over time and after a decade in the county migrants will have a greater psychiatric care use comparable to the Swedish-born population.

## Methods

### Study design

Using data from Psychiatry Sweden (https://ki.se/en/phs/psychiatry-sweden-the-register-linkage-epicss-group), a database of Swedish population and health registers linked for psychiatric research, a longitudinal national population-based cohort study was carried out. Anonymised medical, migration and socioeconomic data were analysed. Data were linked through Swedish personal identity number, assigned to all those born in Sweden and those born abroad living in Sweden with valid residence permits.

### Study population

The study population which comprised individuals born from 1 January 1944 to 31 December 1990, living in Sweden in 2016 and resident in Sweden during 2005–2016 was followed from 1 January 2005 to 31 December 2016. Included were all Swedish-born individuals with two Swedish-born parents (‘Swedish Born’) and all individuals born outside of Sweden from 1944 to 1990, who moved to Sweden prior to 1 January 2005 and were resident in Sweden every year from 2005 to 2016 (‘Migrants’). Excluded were Swedish born with one or two parents born abroad and those without an official residence permit in Sweden—that is, undocumented migrants or individuals awaiting an official asylum decision.

### Data sources

Data were retrieved from the following registers: the Register of the Total Population^19^ was used for extracting information on participants’ birth date, sex, country of birth (grouped in register into regions) and years of residence in Sweden; the Multi-Generation Register^20^ was used to link children to parents within the study; and the longitudinal database for studies of immigrants’ integration^21^ provided migration data. Data on sociodemographic disposable income were taken from the Longitudinal Integration Database for health insurance and labour market studies.^19^ Data on participants’ psychiatric history were taken from the National Patient Register,^22^ which contains complete inpatient psychiatric care records during follow-up.^19^ Data on prescription of psychotropic medication came from the Prescribed Drug Registry,^23^ which contains national information from July 2005.

### Variables

#### Outcome

Psychiatric care use was studied as a combined variable (called *any psychiatric care*) and defined as receipt of the first time use of any psychiatric services during the study period from 2005 to 2016 and coded as binary. Disaggregated psychiatric care use was also studied: *inpatient care* (use of hospital psychiatric services), *outpatient care* (community psychiatric services) and *prescribed and purchased psychotropic medication* including psychotropic prescriptions were categorised into antidepressants (Anatomic Therapeutic Chemical (ATC) code N06A), attention-deficit/hyperactivity disorder medications (ATC N06BA01–No6BA04, N06BA09), antipsychotics (ATC N05A), anxiolytics (ATC N05A) and sedatives/hypnotics (ATC N05C).

#### Explanatory variable

##### Swedish born and migrants

The population was grouped into five categories: Swedish born with two Swedish-born parents and migrants grouped by year of immigration to Sweden: before 1990, 1990–1994, 1995–2004 and 2005 onwards.

##### Region of origin

As a secondary exposure, we classified people according to region of origin, as defined by country of birth. To prevent misuse of data, Statistics Sweden does not record ethnicity or religion, and aggregates country of origin of migrants to Sweden into non-specific regions. Although Statistics Sweden records data on specific country of birth, information is released for research purposes according to 13 larger geographical regions to ensure confidentiality. From this variable, we derived a broader region of origin variable for analysis, which included Sweden (Swedish born with Swedish-born parents) and seven other regions:

- Sub-Saharan Africa.
- Asia.
- Eastern Europe, Russia and the Baltic states.
- Western and Southern Europe.
- The Nordic countries.
- The Middle East and North Africa.
- USA, Canada and Oceania.
- South America.

#### Covariates

We included year of birth and sex as a priori confounders.

### Statistical analysis

Data were analysed in March 2017 to September 2017 and in May and August 2019. Table 1 presents basic descriptive statistics for Swedish born and migrants (stratified on the year of migration: before 1990, 1990–1994, 1995–2004, 2005 and onwards). To examine the effect over time, we implemented a time-stratified analysis, where we examined the cross-sectional effect (using logistics regression) and also survival-based designs (estimating the time-to-failure effect using Cox regression).

**Table 1:** Population characteristics

Immigration period	Swedish born	Migration before 1990	Migration 1990– 1994	Migration 1995– 2004	Migration 2005 onwards	P value*
Total		4 025 413	347 280	162 666	235 768	379 626	
Sex	Female	2 067 781 (51.4%)	168 615 (48.6%)	81 207 (49.9%)	112 655 (47.8%)	199 783 (52.6%)	<0.001
	Male	1 957 632 (48.6%)	178 665 (51.4%)	81 459 (50.1%)	123 113 (52.2%)	179 844 (47.4%)	
Age at start, mean (SD)		38.6 (13.5)	44.0 (11.5)	35.2 (11.6)	32.7 (9.9)	31.6 (9.5)	<0.001
Age at end†, mean (SD)		48.5 (14.0)	53.1 (12.1)	44.1 (12.1)	41.1 (10.7)	38.0 (9.9)	<0.001
Region of origin	Sweden	4 025 413 (100.0%)					
	Sub-Saharan Africa		11 584 (3.3%)	14 166 (8.7%)	17 585 (7.5%)	39 390 (10.4%)	
	Asia		34 295 (9.9%)	13 680 (8.4%)	33 238 (14.1%)	72 251 (19.0%)	
	Eastern Europe, Russia and the Baltics		57 303 (16.5%)	71 559 (44.0%)	54 641 (23.2%)	87 669 (23.1%)	
	Western and Southern Europe		28 833 (8.3%)	4824 (3.0%)	24 195 (10.3%)	41 462 (10.9%)	
	Nordics		112 233 (32.3%)	6175 (3.8%)	22 174 (9.4%)	32 127 (8.5%)	
	Middle East and North Africa		67 702 (19.5%)	43 259 (26.6%)	65 708 (27.9%)	83 442 (22.0%)	
	USA, Canada and Oceania		4813 (1.4%)	1714 (1.1%)	6702 (2.8%)	9379 (2.5%)	
	South America		30 517 (8.8%)	7289 (4.5%)	11 525 (4.9%)	13 907 (3.7%)	
Income quintile‡	Lowest	225 266 (5.6%)	49 223 (14.2%)	45 737 (28.1%)	103 935 (44.1%)	232 315 (61.2%)	<0.001
	Lower middle	748 309 (18.6%)	84 489 (24.3%)	51 515 (31.7%)	62 019 (26.3%)	76 774 (20.2%)	
	Middle	1 137 605 (28.3%)	87 888 (25.3%)	38 123 (23.4%)	36 829 (15.6%)	39 742 (10.5%)	
	Higher middle	1 178 546 (29.3%)	79 859 (23.0%)	20 643 (12.7%)	21 195 (9.0%)	20 158 (5.3%)	
	Highest	735 687 (18.3%)	45 821 (13.2%)	6648 (4.1%)	11 790 (5.0%)	10 638 (2.8%)	
Any psychiatric care	No	3 054 595 (75.9%)	236 346 (68.1%)	111 541 (68.6%)	178 566 (75.7%)	329 800 (86.9%)	<0.001
	Yes	970 818 (24.1%)	110 934 (31.9%)	51 125 (31.4%)	57 202 (24.3%)	49 827 (13.1%)	
Inpatient care	No	3 897 475 (96.8%)	332 476 (95.7%)	156 838 (96.4%	228 948 (97.1%)	373 804 (98.5%)	<0.001
	Yes	127 938 (3.2%)	14 804 (4.3%)	5828 (3.6%)	6820 (2.9%)	5823 (1.5%)	
Outpatient care	No	3 632 448 (90.2%)	303 479 (87.4%)	140 846 (86.6%)	211 977 (89.9%)	361 080 (95.1%)	<0.001
	Yes	392 965 (9.8%)	43 801 (12.6%)	21 820 (13.4%)	23 791 (10.1%)	18 547 (4.9%)	
Prescribed medication	No	3 157 534 (78.4%)	247 281 (71.2%)	116 942 (71.9%)	185 242 (78.6%)	336 528 (88.6%)	<0.001
	Yes	867 879 (21.6%)	99 999 (28.8%)	45 724 (28.1%)	50 526 (21.4%)	43 099 (11.4%)	

*χ^2^ test for categorical variables and t-test for continuous variables.

†It is specified with the event as any psychiatric care.

‡The mean income category over the individual’s follow-up period.

**Figure 1:**
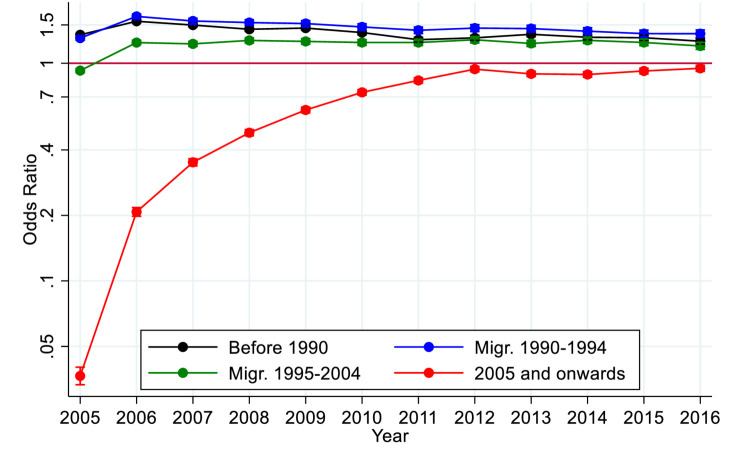
ORs and 95% CI for the first time use of any psychiatric care during time of follow-up among all migrants to Sweden by year of immigration: before 1990, 1990–1994, 1995–2004 or 2005 onwards, compared with Swedish born.

In our first analysis (figure 1) we estimated the effect the different migration periods had compared with Swedish born with regard to the use of any psychiatric care, within each year, from 2005 to 2016, using logistic regression. We adjusted for sex and year of birth, and displayed graphically the ORs along with their 95% CIs. All within-strata models were estimated using robust SEs.

**Figure 2:**
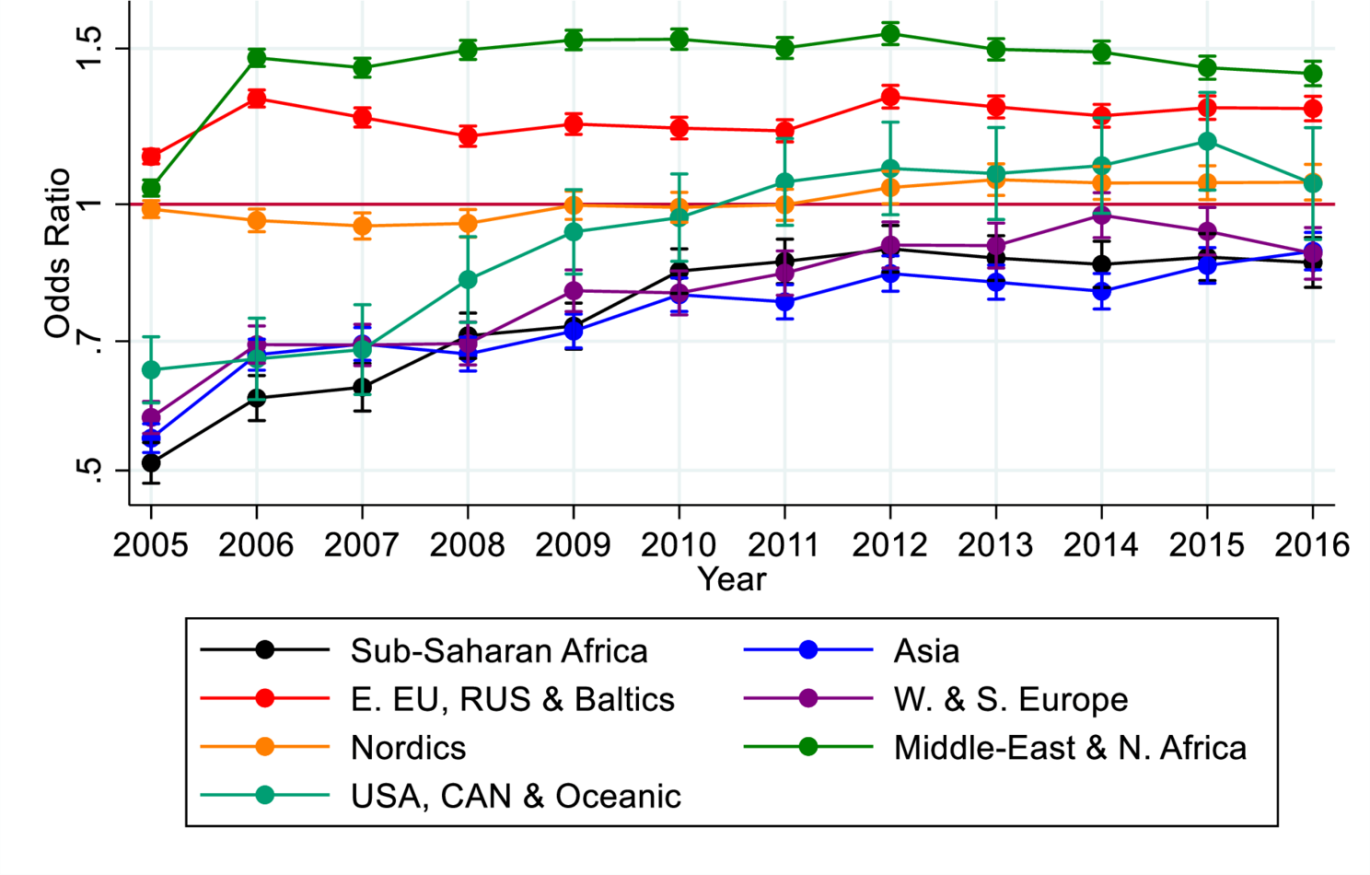
OR and 95% CI for the first time use of psychiatric care during time of follow-up among all migrants to Sweden by region of origin: sub-Saharan Africa; Asia; Eastern Europe, Russia and the Baltics; Western and Southern Europe; Nordics; Middle East and North Africa; USA, Canada and Oceania; and South America, compared with Swedish born.

The second analysis (figure 2) was similar but instead examined the effect of regions of origin on use of any psychiatric care. The adjustments were still year of birth and sex, with a graphical display of the OR over time, followed by the reported 95% CI which were estimated with a robust SE. The sex-stratified analyses can be found in online supplementary figures C and D.

10.1136/bmjgh-2020-002471.supp1 Supplementary data

**Figure 3:**
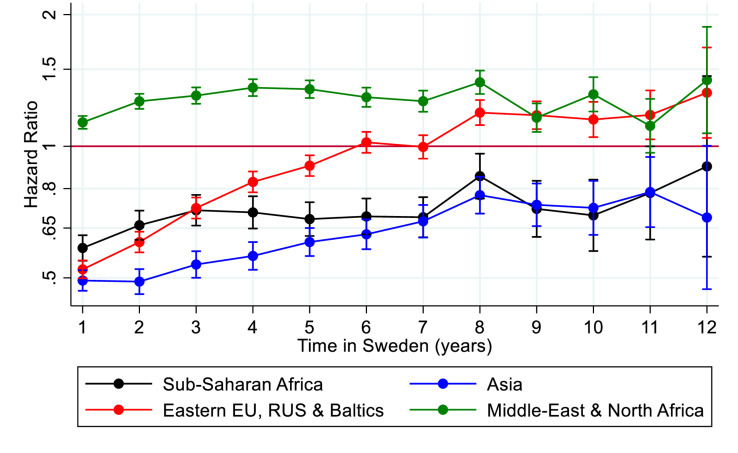
HR and 95% CI for the first time use of psychiatric care during time to follow-up among migrants to Sweden by region of origin: sub-Saharan Africa; Asia; Eastern Europe, Russia and the Baltic states; and Middle East and North Africa compared with Swedish born.

Within-year survival analyses were conducted, with the underlying timescale ‘Time in Sweden’. We estimated the time until use of any psychiatric care, based on migrants who immigrated after 2005, categorised regions of origin with Sweden as the reference category (figure 3). Swedish born’s time at risk was considered to have started on their entry into the study, for comparison purposes.

**Figure 4:**
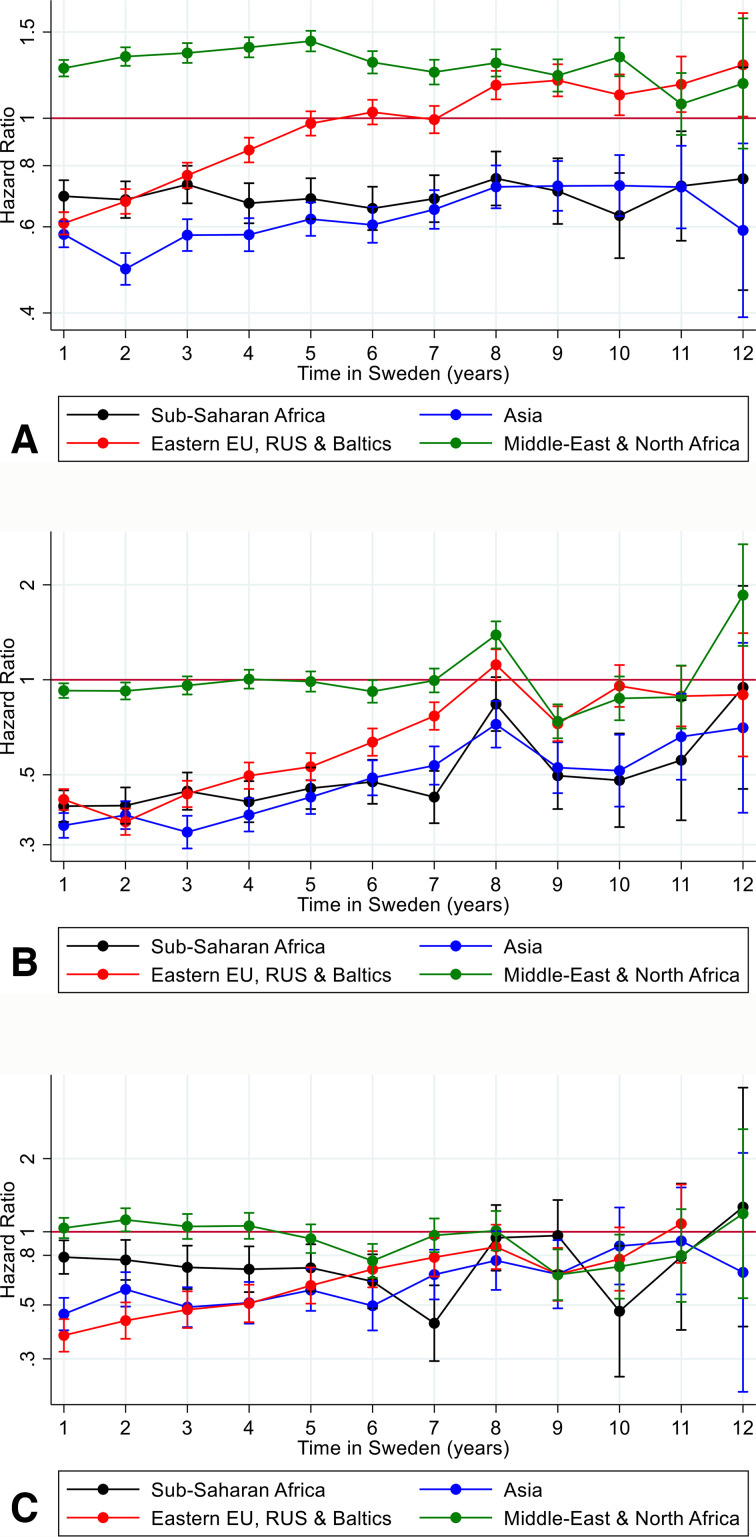
HR and 95% CI for the first time use of psychiatric care during time of follow-up among migrants to Sweden for migrants from sub-Saharan Africa; Asia; Eastern Europe, Russia and the Baltic states; and Middle East and North Africa compared with Swedish born by type of psychiatric care medication, inpatient care, outpatient care and all care.

To examine a possible bias towards migrants using different psychiatric treatments based on their region of origin, survival analysis was re-estimated based on the specific psychiatric outcomes: prescribed psychotropic (figure 4A), outpatient care (figure 4B) and inpatient care (figure 4C).

In the sensitivity analyses, we examined whether there was a difference between men and women (online supplementary figures A–D). While we did find marginal differences between the sexes, the patterns of the development remained consistent throughout the individuals’ study period.

Due to the unexpected pattern of migrants from the Middle East and North Africa (after 2005 migration), we decided to do a post hoc in-depth of this specific group (online supplementary figure E).

## Results

Our final cohort consisted of 5 150 753 individuals (table 1), where 78.1% were Swedish born. Sex composition was similar across groups, although the Swedish born and the latest coming group (migration from 2005) and onwards had a small majority of women. Migrants had lower disposable income than Swedish born, with 61.2% of migrants arriving since 2005 in the lowest income quintile. The largest group migrating to Sweden before 1990 was from the Nordic countries. The largest group migrating to Sweden from 1990 to 1994 was from Europe, Russia and the Baltic states. The largest group migrating to Sweden in 1995 and onwards was from Eastern Europe and the Middle East (table 1).

Migrants who arrived in Sweden before 1990, or between 1990 and 1994, and 1995 and 2004, all had a significantly higher use of any psychiatric care, as compared with Swedish born (see figure 1). Migrants who arrived in Sweden in 2005 onwards had significantly lower use of psychiatric care during follow-up, however the differences as compared with the Swedish reference group decreased over time (see figure 1). The pattern was the same for both men and women (see online supplementary figure A, B).

There were differences in any psychiatric care utilisation among all migrants by region of origin too (see figure 2). Migrants from sub-Saharan Africa, Asia, and Western and Southern Europe had lower use of any psychiatric care during follow-up. Migrants from the USA, Canada and Oceania had a lower utilisation during the first years of follow-up but higher towards the end. Migrants from the Nordic countries were similar to the Swedish referent group. Migrants from the Middle East and North Africa, and Eastern Europe, Russia and the Baltic states had a higher utilisation. This pattern was similar for both women and men (see online supplementary figure C and D).

As migrants who arrived in Sweden in 2005 had significantly lower utilisation as compared with the other groups, the following analyses focus on this group and the migrants from the region of sub-Saharan Africa, Asia, Eastern Europe, Russia and the Baltic states, and Middle East and North Africa. The same analyses were made for the other years of immigration to Sweden (before 1990, 1990–1994, 1995–2004) and for the other migrant groups, and can be requested from the authors. Migrants from sub-Saharan Africa and Asia had a significantly lower utilisation of psychiatric care during the first 11 years in Sweden (see figure 3). Migrants from Eastern Europe, Russia and the Baltics started at a lower level than the Swedish born but after 5 years in Sweden they had an equal likelihood of using psychiatric care. Migrants from Middle East and North Africa were the only group of migrants with a significantly higher utilisation of psychiatric care from immigration and onwards. We studied the migrant group from the Middle East and North Africa by countries and groups of countries included and found that Iran and Iraq both had a higher psychiatric healthcare utilisation than Swedish born and that migrants from the rest of the Middle East and North Africa had psychiatric healthcare utilisation similar to Swedish born (see online supplementary figure E).

We also studied psychiatric care utilisation for migrants from sub-Saharan Africa, Asia, Eastern Europe, Russia and the Baltic states, and Middle East and North Africa during their first decade in Sweden by type of psychiatric care (see figure 4A–C). It turned out the higher use of psychiatric care among migrants from the Middle East and North Africa was driven by use of prescribed and purchased and psychotropic medication, and for the groups with a lower use of any psychiatric care utilisation the use of prescribed and purchased psychotropics was more similar to the Swedish patterns than inpatient and outpatient psychiatric care (see figure 4C). Low levels of outpatient care were driving the low levels of psychiatric care in the migrant groups with low levels during the first decade in Sweden.

## Discussion

### Statement of principal findings

This study investigates differences in patterns of psychiatric care use between Swedish born and all migrants to Sweden; and determines how these patterns are influenced by time in Sweden, region of origin and type of care. In line with our hypothesis, most migrants to Sweden, both men and women, typically underuse psychiatric services during the first decade in Sweden. However, use increases with time, and contrary to our hypothesis, those who have been in Sweden for more than a decade typically use more psychiatric care than Swedish born. Migrant groups with a particularly low use during the first years included those from sub-Saharan Africa, Asia, and Western and Southern Europe. The lower psychiatric care use among migrants compared with the Swedish born during the first decade was explained by lower use of specialist outpatient services. Contrary to our hypothesis, not all migrants have a lower psychiatric care use than Swedish born during their first decade in Sweden, as migrants from the Middle East and North Africa who had arrived in Sweden after 2005 had a higher psychiatric care utilisation since start of follow-up, though this was driven by a higher use of psychotropic medication.

### Strengths and weaknesses of the study

To our knowledge, this is the largest study to date of migrant psychiatric care use^2^ using comprehensive psychiatric care outcomes (psychotropics, outpatient and inpatient care). Our study is strengthened by the use of Swedish register data, which are high quality,^22^ and inclusion of data on sex and region of origin implemented in a within-time stratified survival analysis. A strength of our data is the long follow-up, 2005–2016, although we should be mindful that studies with longer follow-up periods have the potential to introduce biases which may arise due to changes in the healthcare system during the time (such as in this time period in Sweden, subspecialised psychiatric clinics, and national and regional guidelines of care for specific diagnoses). Nonetheless, we have no reason to believe such changes are differential and would have acted differentially by migrant status. Lacking from our study are direct measures of mental illness (ie, diagnoses) and as such low utilisation by a group may indicate good mental health, impediments to accessing care or seeking healthcare outside the Swedish state. This could possibly be an explanation for migrants from Western and Southern Europe, and USA, Canada and Oceania as these are primarily labour migrants known for their better mental health^24^ and possibilities to psychiatric care in their countries of origin; however, for the other migrants this explanation seems unlikely due to a high prevalence of poor mental health.^1–3^ Register-based data are collected routinely as measures of healthcare utilisation, and this does not include other forms of help seeking such as seeking care in country of origin, traditional or complementary medicine. Register data grouping prevents most analysis by country of origin, and as such disparate ethnic, cultural and national groups may be inappropriately grouped. Although this study attempts to control for age, age at migration, region of origin and time in Sweden, it is difficult to disentangle the differences in sequential waves of migration from different origin contexts and associated demographics.

### Strengths and weaknesses in relation to other studies

It has been noted by previous studies that migrants have lower psychiatric care utilisation compared with majority populations,^2^ and lower rates of consultation in primary care settings for mental health-related problems.^6^ Our results show a clear shift in psychiatric care use after 10 years. Our study finds that length of stay in a country is a crucial factor in terms of psychiatric care utilisation and that after 10 years the general profile of care use approaches that of Swedish born. Interestingly, migrants from the Middle East and North Africa were different from the other migrant groups as they displayed high psychiatric utilisation from beginning of follow-up. In a recent Norwegian study, Iraqi men in Norway, contrary to all other migrant groups, had a higher use of psychiatric care.^6^ Another Norwegian study found that migrants from Iran and Iraq, also contrary to all other migrants, had higher utilisation of psychiatric care.^5^ Migrants from sub-Saharan Africa displayed a particularly low likelihood of any psychiatric care in the first 10 years and throughout the study. In the UK, black minority populations are less likely to be taking antidepressants than the white majority population even after adjusting for symptom severity.^7^ In the same study, black and South Asian minority groups were less likely to have contacted a general practitioner about their mental health in the last year. However, in many studies people from a black ethnic minority background have an increased risk of emergency care at first contact^9 10 25^ or coercive care.^26^ In our study, psychotropics explained those instances where migrants had a higher use during the first 10 years and this is seen in an older previous study from Sweden using cross-sectional survey data.^8^

### Meaning of the study

The lower use of psychiatric care among migrants during the first decade in the country may indicate that the barrier to psychiatric care is lowered over time in Sweden. As studies show that migrants to high-income countries have a higher prevalence of some specific psychiatric diagnoses, such as PTSD and psychosis during these first years,^1–3^ the latency to reach comparable usage to Swedish born is concerning, particularly for serious mental conditions. The higher use of prescribed and purchased psychotropics among migrants from the Middle East and North Africa is also seen in other studies, such as a similar record linkage study from Finland.^27^ However, this group had a lower use of antidepressant in a study from Northern Ireland.^28^ Prescribed and purchased psychotropics do not necessarily equate to inpatient or outpatient psychiatric care. The former indicates that while prescribing physicians may identify patients from the Middle East and North Africa as needing of mental health treatment, these patients, for reasons which remain unknown, do not receive inpatient or outpatient care. This is unlikely to be attributed to the share of refugees in these groups, as there are similar large shares of refugees among migrants from sub-Saharan Africa who display different trends of care use. It is possible that migrants from Middle East/North Africa have health-seeking behaviours towards psychotropic interventions; however, it may also represent an imposition by the health system regarding providing the relevant healthcare for these minority groups.

Another key result is that longer established migrants in Sweden have higher use of psychiatric care, implying that barriers to psychiatric care are lowered over time, suggesting a possible acculturation to the Swedish psychiatric healthcare system. Low initial use of psychiatric care use across most migrant groups may indicate that these groups have lower overall rates of mental illness in recent migrants (PTSD and psychosis notwithstanding), and hence a lower need of psychiatric care (the ‘healthy migrant effect’). Our group has previously shown that immigrants to Sweden have lower rates of both substance use disorders and suicide,^29 30^ for example, although rates converge to the Swedish baseline rate over time. This ‘healthy migrant effect’ may explain patterns of care use observed driven by labour migration, and associated with groups have previously demonstrated better general mental health.^24^ However, studies also show that migrants to Sweden display high levels of mental health issues (other than substance use and suicide) during the first years of migration, hence underutilisation is a more likely explanation.^31–35^ Migrants from the Nordic countries have similar patterns of psychiatric care to the Swedish born, possibly highlighting the importance of both language and cultural similarities and similarity and familiarity of healthcare systems between those in a migrant’s country of origin and a destination country in which they settle, which may lower barriers to care. This has also been highlighted in a qualitative study of Polish workers in Northern Ireland.^36^

### Unanswered questions and future research

More qualitative research is needed to investigate migrants’ experience of psychiatric services, and care providers’ differential treatment of immigrant and ethnic minority populations. Research on structural barriers to care is essential to further develop early intervention inclusive services and prevent deleterious mental health trajectories in vulnerable populations. Other important research needed is to link diagnoses with access to care, to address the limitation of most studies that look at utilisation without a needs assessment.

### Patient and public involvement

No patients were involved in setting the research question or the outcome measures, nor were they involved in developing plans for design or implementation of the study. No patients were asked to advise on interpretation or writing up of results. However, we will disseminate the results of our research to agencies responsible for the healthcare of migrants in Sweden.
