# Supplementary material for: Psychiatric care use among migrants to Sweden compared with Swedish-born residents: a longitudinal cohort study of 5 150 753 people
Source: BMJ Glob Health. 2020 Sep 24;5(9):e002471. doi: 10.1136/bmjgh-2020-002471 (PMC7517566; doi:10.1136/bmjgh-2020-002471)

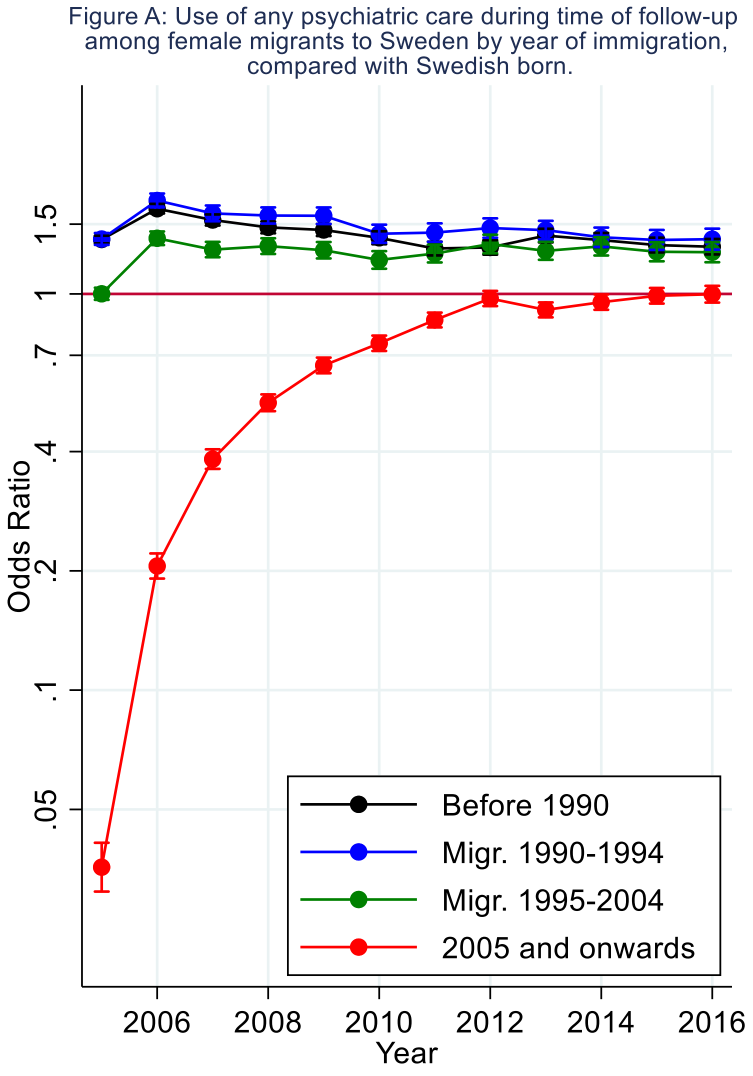
Figure A: Use of any psychiatric care during time of follow-up among female migrants to Sweden by year of immigration, compared with Swedish born.
Odds Ratio
.05
.1
.2
.4
.7
1
1.5
2006
2008
2010
2012
2014
2016
Year
Before 1990
Migr. 1990-1994
Migr. 1995-2004
2005 and onwards

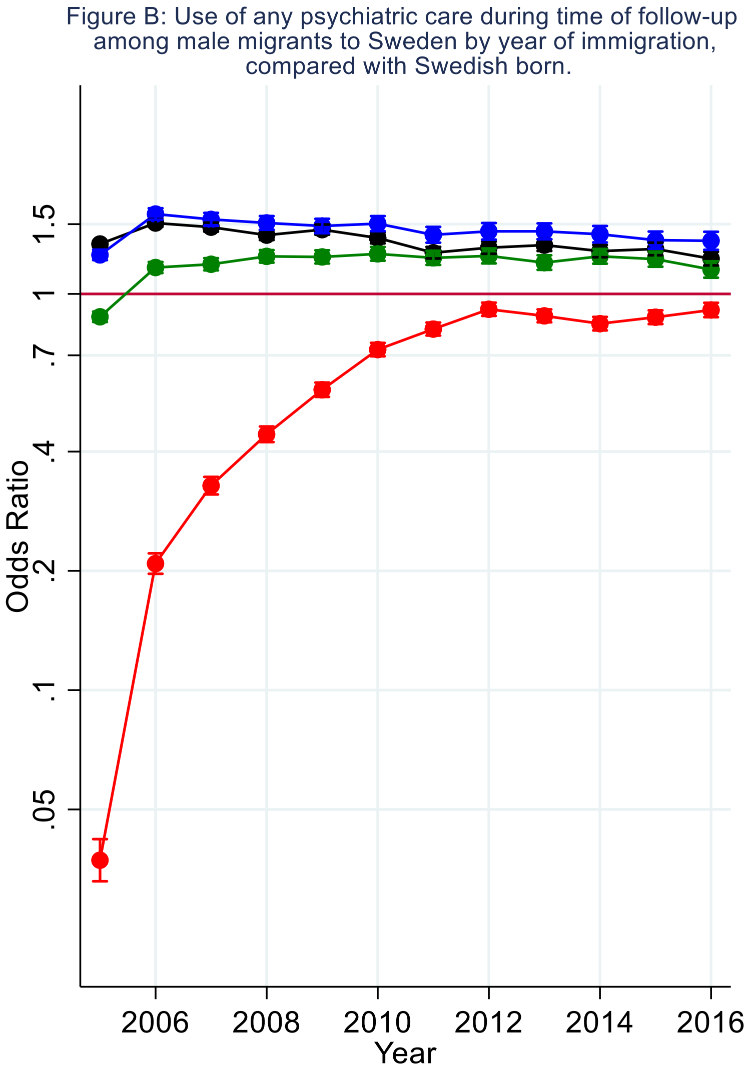
Figure B: Use of any psychiatric care during time of follow-up among male migrants to Sweden by year of immigration, compared with Swedish born.
Odds Ratio
.05
.1
.2
.4
.7
1
1.5
2006
2008
2010
2012
2014
2016
Year

Figure C: Any psychiatric care during time of follow-up among female migrants to Sweden by region of origin, compared to Swedish born.

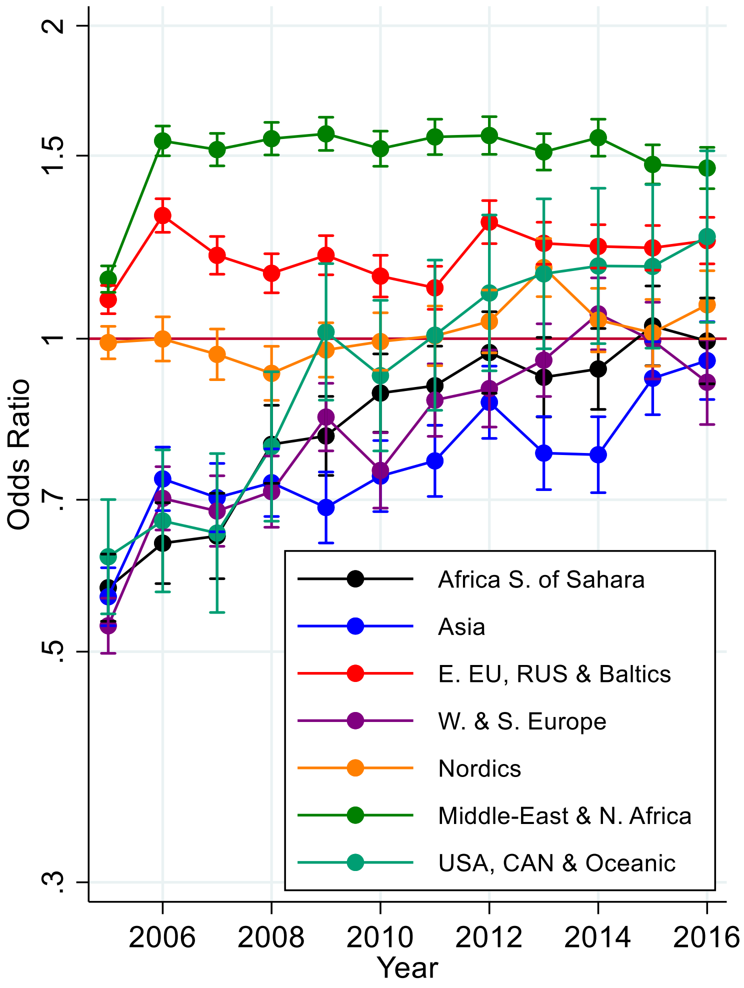

Figure D: Any psychiatric care during time of follow-up among male migrants to Sweden by region of origin, compared to Swedish born.

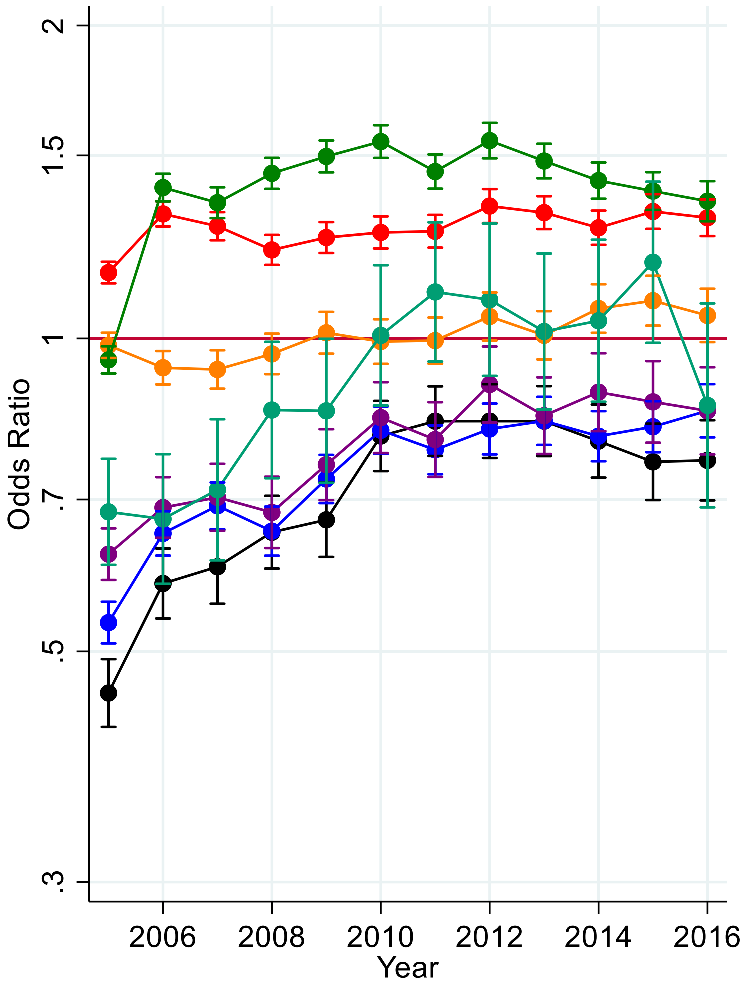

Figure E: Any psychiatric care during time of follow-up among migrants to Sweden immigrating from the Middle-East and North Africa from 2005 and onwards from four regions/countries, compared with Swedish-born.

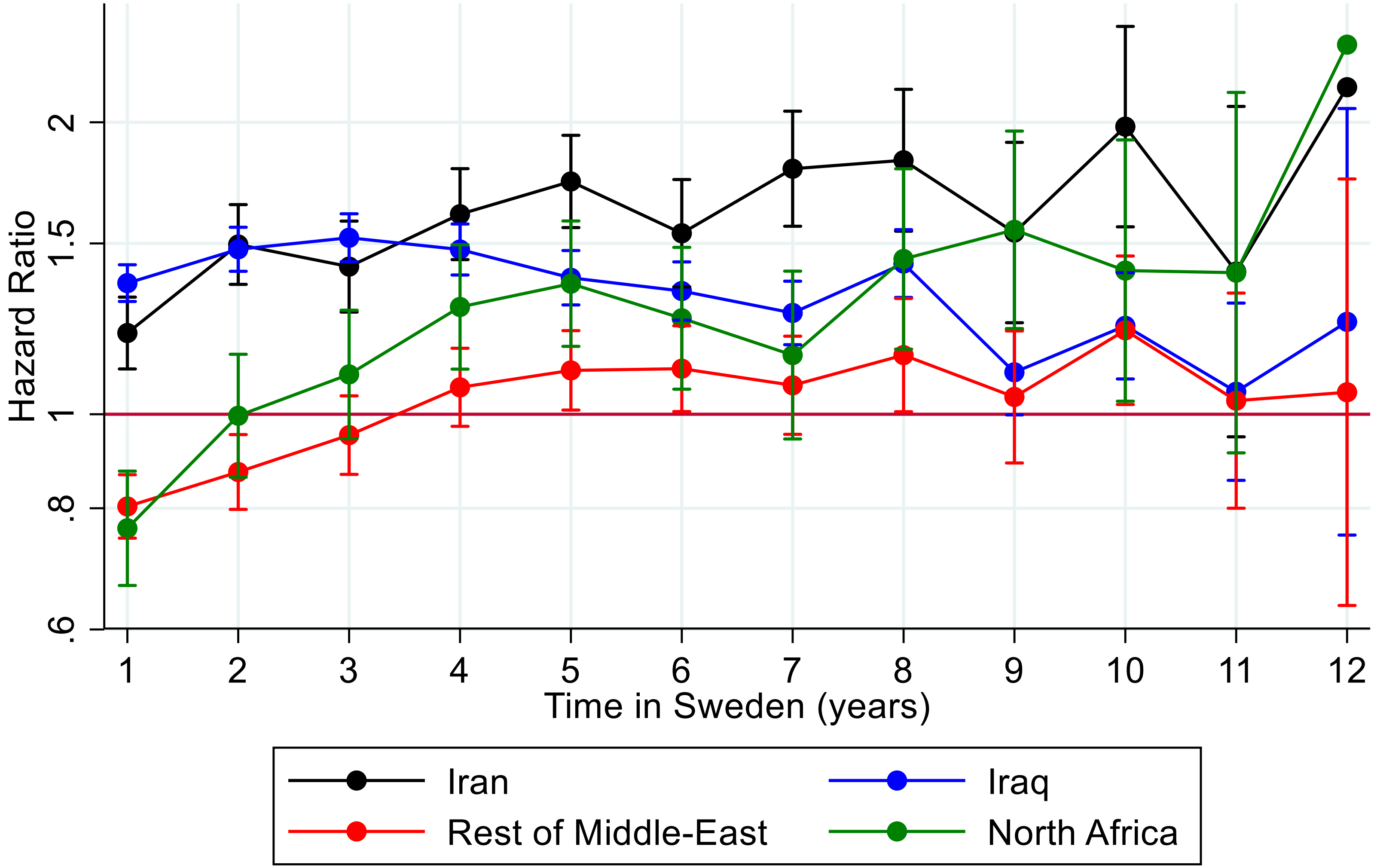

Supplement: Supplementary data [file bmjgh-2020-002471supp001.pdf]
